# Paediatric Medicines in Europe: The Paediatric Regulation—Is It Time for Reform?

**DOI:** 10.3389/fmed.2021.593281

**Published:** 2021-02-02

**Authors:** Maddalena Toma, Mariagrazia Felisi, Donato Bonifazi, Fedele Bonifazi, Viviana Giannuzzi, Giorgio Reggiardo, Saskia de Wildt, Adriana Ceci, TEDDY European Network of Excellence for Paediatric Research

**Affiliations:** ^1^Fondazione per la Ricerca Farmacologica “Gianni Benzi” Onlus, Bari, Italy; ^2^Consorzio per Valutazioni Biologiche e Farmacologiche, Bari, Italy; ^3^Radboud Institute for Molecular Life Sciences, Radboud University Medical Center, Nijmegen, Netherlands

**Keywords:** EU paediatric regulation, paediatric medicines, paediatric age, therapeutic areas, paediatric clinical studies, paediatric repurposing, orphan paediatric medicines

## Abstract

**Objectives:** In this paper, we investigated the effects of the European Paediatric Regulation (EC) N° 1901/2006 with respect to satisfying the paediatric therapeutic needs, assessed in terms of the increased number of paediatric medicinal products, new therapeutic indications in specific high-need conditions (neonates, oncology, rare disease, etc.) and increased number of paediatric clinical studies supporting the marketing authorisation.

**Methods:** We analysed the paediatric medicinal products approved by the European Medicines Agency in the period January 2007-December 2019, by collecting the following data: year of approval, active substance, legal basis for the marketing authorisation, type of medicinal product (i.e., chemical, biological, or ATMP), orphan drug status, paediatric indication, Anatomical Therapeutic Chemical code (first-level), number and type of paediatric studies. Data were compared with similar data collected in the period 1996–2006.

**Results:** In the period January 1996–December 2019, in a total of 1,190 medicinal products and 843 active substances, 34 and 38%, respectively, were paediatric. In the two periods, before and after the Paediatric Regulation implementation, the paediatric/total medicinal products ratio was constant while the paediatric/total active substances ratio decreased. Moreover, excluding generics and biosimilars, a total of 106 and **175 paediatric medicines** were granted a **new paediatric indication, dosage or age group** in the two periods; out of 175, 128 paediatric medicines had an approved Paediatric Investigational Plan. The remaining 47 were approved without an approved Paediatric Investigational Plan, following the provisions of Directive 2001/83/EC and repurposing an *off-patent* drug. The analysis of the clinical studies revealed that drugs with a Paediatric Investigational Plan were supported by 3.5 studies/drug while drugs without a Paediatric Investigational Plan were supported by only 1.6 studies/drug.

**Discussion:** This report confirms that the expectations of the European Paediatric Regulation (EC) N° 1901/2006 have been mainly satisfied. However, the reasons for the limited development of paediatric medicines in Europe, should be further discussed, taking advantage of recent initiatives in the regulatory field, such as the Action Plan on Paediatrics, and the open consultation on EU Pharmaceutical Strategy.

## Introduction

In Europe, children represent more than 20% of the population, with about 100 million people aged <19 years. Notwithstanding this, more than 70% of marketed drugs do not include a paediatric authorisation and havenot been properly tested and presented for the paediatric population (1, 2).

There are several aspects behind the shortage of paediatric medicines. Many issues affect the research and development of children's medicines, including: ethical concerns and difficulties of informed consent and assent management (3), no clear criteria for evaluating the potential risks of children's exposure in a trial, the cost of paediatric clinical trials, which are higher than clinical trials with adults due to the multiple paediatric population to be included (4), challenges in recruitment for paediatric trials, difficulties in trial design (i.e., small numbers of eligible patients and lack of appropriate age-matched controls), etc.

With the aim of handling these concerns and assuring that children have safe access to both old and new medicinal products (MPs), the European Paediatric Regulation (EC) N° 1901/2006 (5) (Paediatric Regulation) entered into force on 26th January 2007.

The Paediatric Regulation established the European Medicines Agency-Paediatric Committee (EMA-PDCO) and made a Paediatric Investigational Plan (PIP) mandatory, prescribing studies in the paediatric population whose results have to be included in the Marketing Authorisation (MA) documentation unless a waiver is granted. It is also possible to grant a deferral in order to delay the results of some studies. These provisions apply to any new or *in patent* drug for which a MA or a MA variation is requested (articles 7 and 8 of the Paediatric Regulation). To compensate for the burden of this requirement, incentives are available to the industry, including a 6-month extension of the supplementary protection certificate and an additional 2 years of market exclusivity for paediatric orphan medicinal products (p-OMPs).

Furthermore, the Paediatric Regulation introduced a new type of MA, the Paediatric Use Marketing Authorisation (PUMA), which is a voluntary procedure, offering 8 plus 2 years of data and market protection to any *off-patent* medicinal product developed for exclusive use in the paediatric population.

The Paediatric Regulation allows exceptions to articles 7 and 8 (6), such that *off patent* products can be granted a MA under Directive 2001/83/EC (7) instead of applying for a PUMA. Directive 2001/83/EC includes provisions relating to generics, biosimilars or hybrid products, as well as well-established active substances for medicinal use and combinations of substances, in case a new indication or other variations are required.

In all these cases, a paediatric marketing authorisation is allowed but a PIP application is not mandatory, and paediatric studies are required case-by-case under the responsibility of the European Medicines Agency's (EMA's) Committee for Medicinal Products for Human Use (CHMP).

Previous reports and studies have described the progress made in Europe in fostering the approval of paediatric medicines after the setting up of the European Medicine Agency and the entering into force of the Paediatric Regulation.

Ceci et al. (8), pointed out the positive effect of the EMEA (now known as the EMA) Centralised Procedure and underlined, in particular, that “under the EMEA centralised procedure, several ASs have been licensed for children. Consequently, serious and life-threatening diseases such as AIDS and diabetes are now treatable” and that “the percentage of paediatric medicines approved in a few years by the EMEA was significantly higher than the percentage of paediatric medicines approved under the National or Mutual Recognition European procedures, (33 vs. to 13.2%).” It concluded supporting the setting up of an EU paediatric initiative similar to that already existing in the Food and Drug Administration (FDA).

In a second paper, Ceci et al. (1), underlined that after 10 years of the EMA Centralised Procedure application, the global percentage of paediatric medicines on the total of MPs was similar (33.2%) to that one showed in the previous report, with a limited number of paediatric MPs (p-MPs) for younger children and therapeutic areas such as neurology and oncology; an increased number of paediatric p-OMPs (56% of the total OMPs) was observed too. The number of medicines with a whole developmental paediatric clinical plan presented at the time of the MA application was also found increased.

More recently other reports and publications (9–14), also recognised how the provisions established by the Paediatric Regulation have been implemented, underling the setting up of the Paediatric Committee and the submission and completion of more than 1.000 PIPs by the end of 2018 (12) with variable percentages across therapeutic areas. Particularly relevant was considered the increased number of marketed paediatric medicines and the high quality of paediatric clinical trials and studies (13).

However, some limitations have been also underlined in these publications and in the analyses done by EMA and the European Commission (EC), i.e., the low coverage of relevant paediatric therapeutic needs (neonates, orphan diseases, neurology), the delay in developing innovative medicines in comparison with the adults innovative MPs, and the very low interest by the sponsors in approaching incentives offered by the Regulation mainly with reference to the PUMA scheme (only six PUMA authorised by the end of 2018) (12).

Moreover, it is to be considered that only paediatric medicines approved under the Paediatric Regulation provisions are included in these papers, reports and evaluation documents, while it would be of interest to consider the whole paediatric medicines framework as evolving in these years. Also, comparisons by different periods are very limited and specifically included in only one publication (13).

The aim of this report is to analyse the pattern of the paediatric medicines approved by the EMA, assessed in terms of the rate of increase of paediatric medicinal products (p-MPs) compared to total, annual increase of approved new paediatric medicines, new therapeutic indications in specific high-need therapeutic areas (neonates, oncology, rare diseases, etc.), and the number and completeness of paediatric clinical studies supporting the MA.

## Materials and Methods

### Sample

The study sample consisted of p-MPs approved by the EMA in the period 26 January 2007–31 December 2019. p-MPs are MPs that include a therapeutic indication for one or more paediatric ages (from birth to <18 years) in the Summary Product Characteristics and Package Leaflet, and/or a specified dosage by age. This encompassed any p-MP's first approval and any paediatric variation of a MP that was already marketed. Comparison was made with p-MPs approved by the EMA in the period 1996–2006.

### Source

The search for EMA paediatric medicines was performed on the European Paediatric Medicines Database (EPMD) (15), managed by TEDDY—European Network of Excellence for Paediatric Research. The EPMD gathers data on p-MPs receiving a centralized MA since 1996, deriving information from EMA official sources (16). The search was performed in the period January 2020–February 2020.

### Data Collected

For each p-MP, the following data were considered: year of approval, active substance (AS), legal basis for the MA submission; type [chemical, biological or Advanced Therapy Medicinal Product (ATMP)], orphan drug status, paediatric indication, age for which the drug is intended, Anatomical Therapeutic Chemical (ATC) code (first-level), number and type of paediatric studies included in the marketing authorisation package [i.e., Pharmacokinetics/Pharmacodynamics (PK/PD), efficacy/safety, other studies including observational, extrapolation, modelling, and simulation].

### Data Analysis and Statistical Methods

The total number and the annual estimated increase rate of paediatric/not-paediatric MPs and ASs were considered for the whole period and for the period before and after the Paediatric Regulation entered into force. A linear regression method was used for the longitudinal analysis of each dependent variable. The analysis of covariance (ANCOVA) was proposed to compare the regression lines: if the *p-*value of the interaction was <0.05, the two slopes were different, and a one unit change in the time (year) was associated with a different mean chance in the response variable.

Differences, in terms of new paediatric indication, age groups, orphan indication, number and type of paediatric studies, were analysed in two groups—p-MPs approved under the Paediatric Regulation and p-MPs approved outside the Paediatric Regulation– and described using both descriptive and inferential statistics.

## Results

### EMA Approved MPs/ASs

**Figure 1:**
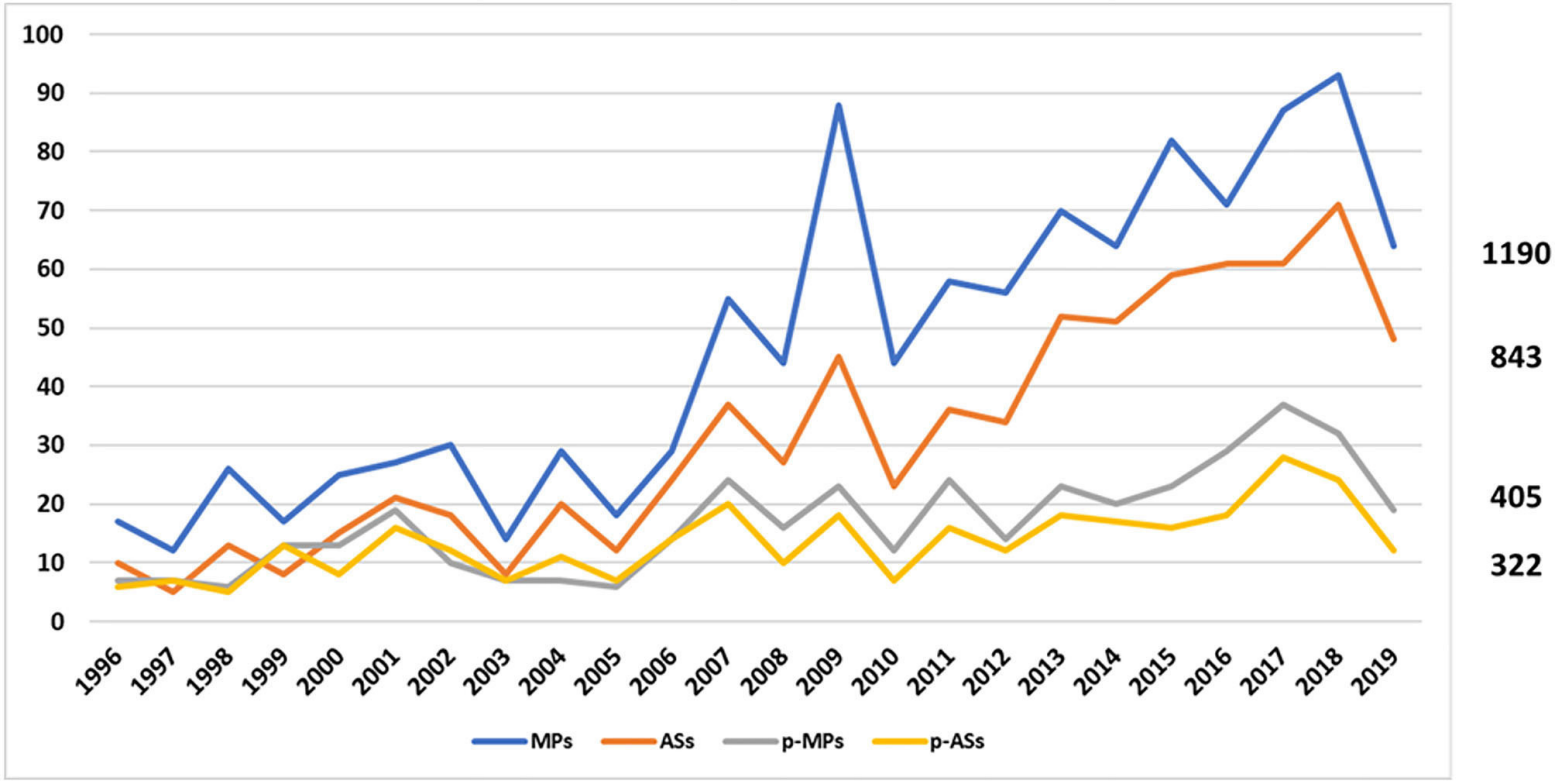
Trend of EMA medicines from January 1996 to December 2019. The blue line represents the medicinal products, the orange one the active substances, the grey one the paediatric medicinal products, and the yellow one the paediatric active substances.

In December 2019, with the exclusion of withdrawn products, a total of **1190 MPs**, corresponding to **843 ASs**, were on the market in Europe, authorised under the Centralised Procedure. Of these, **405 MPs** (34%), corresponding to **322 ASs** (38%), were also approved for children (Figure 1).

More specifically, during the period 2007–2019, 296 MPs (34%) and 216 ASs (36%) were approved as paediatric medicines, demonstrating that the p-MPs/MPs ratio remained stable while the p-ASs/ASs ratio decreased compared to the previous period (1996–2006) (Table 1).

**Table 1:** Number of p-MPs and p-ASs before and after the Paediatric Regulation.

	**1996–2006**	**2007–2019**	**Total**
TOTAL MPs	314	876	1,190
TOTAL p-MPs	109	296	405
p-MPs/MPs ratio	35%	34%	34%
TOTAL ASs	238	605	843
TOTAL p-ASs	106	216	322
p-ASs/ASs ratio	45%	36%	38%

#### Increase Rate of Approved MPs/ASs

**Figure 2:**
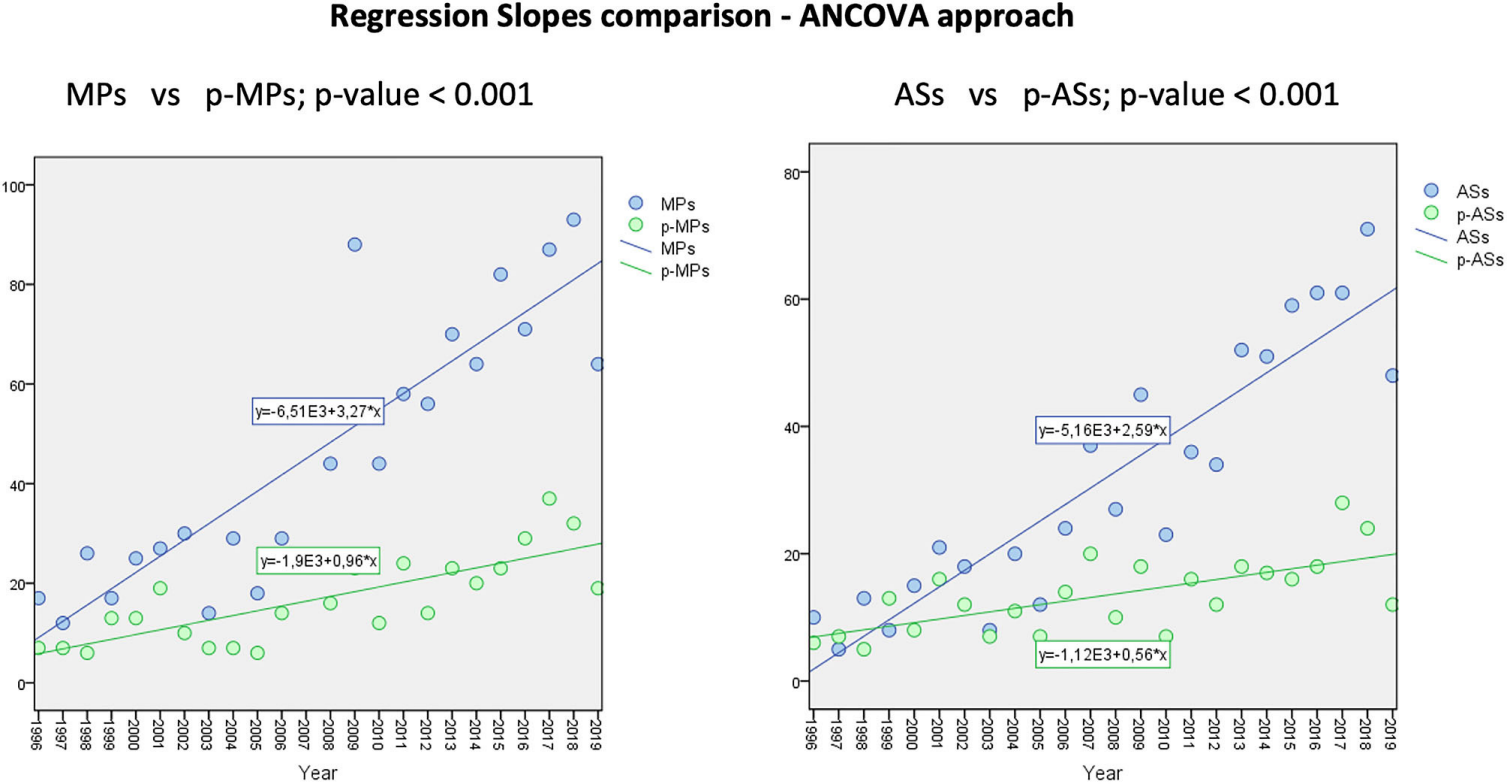
Medicinal Products and Active Substances increase rate from 1996 to 2019.

The annual trend for increase is shown in Figure 2 and demonstrates a relevant increase of MPs approved by the EMA since the set-up of the Agency, while the average annual increase of both p-MPs and p-ASs is significantly lower.

### Regulatory Details of p-MPs and p-ASs

Of a total of 296 p-MPs (corresponding to 216 p-ASs), 136 (130 ASs; 45.9%) were approved following the provisions of the Paediatric Regulation after having submitted a PIP; of these, only 27 had completed the PIP at the time of approval.

The remaining 160 p-MPs (86 ASs; 54.1%) were approved according to Directive 2001/83/EC without submitting a PIP.

#### Exceptional MA

A total of 33 paediatric drugs underwent a fast track approval (exceptional circumstances, conditional approval, accelerated assessment), 9 in the no-PIP group. In both groups, anticipated MAs have been granted mainly in case of OMPs for inborn errors of metabolism, followed by blood and oncology indications.

#### Additional Monitoring

The number of paediatric medicines licensed with the request of receiving an additional monitoring was very high (67/136 (49%) in the PIP group and less in the no-PIP group. No mention of the follow up of these studies is included in any official data sources.

### New Paediatric Medicines Characteristics

**Figure 3:**
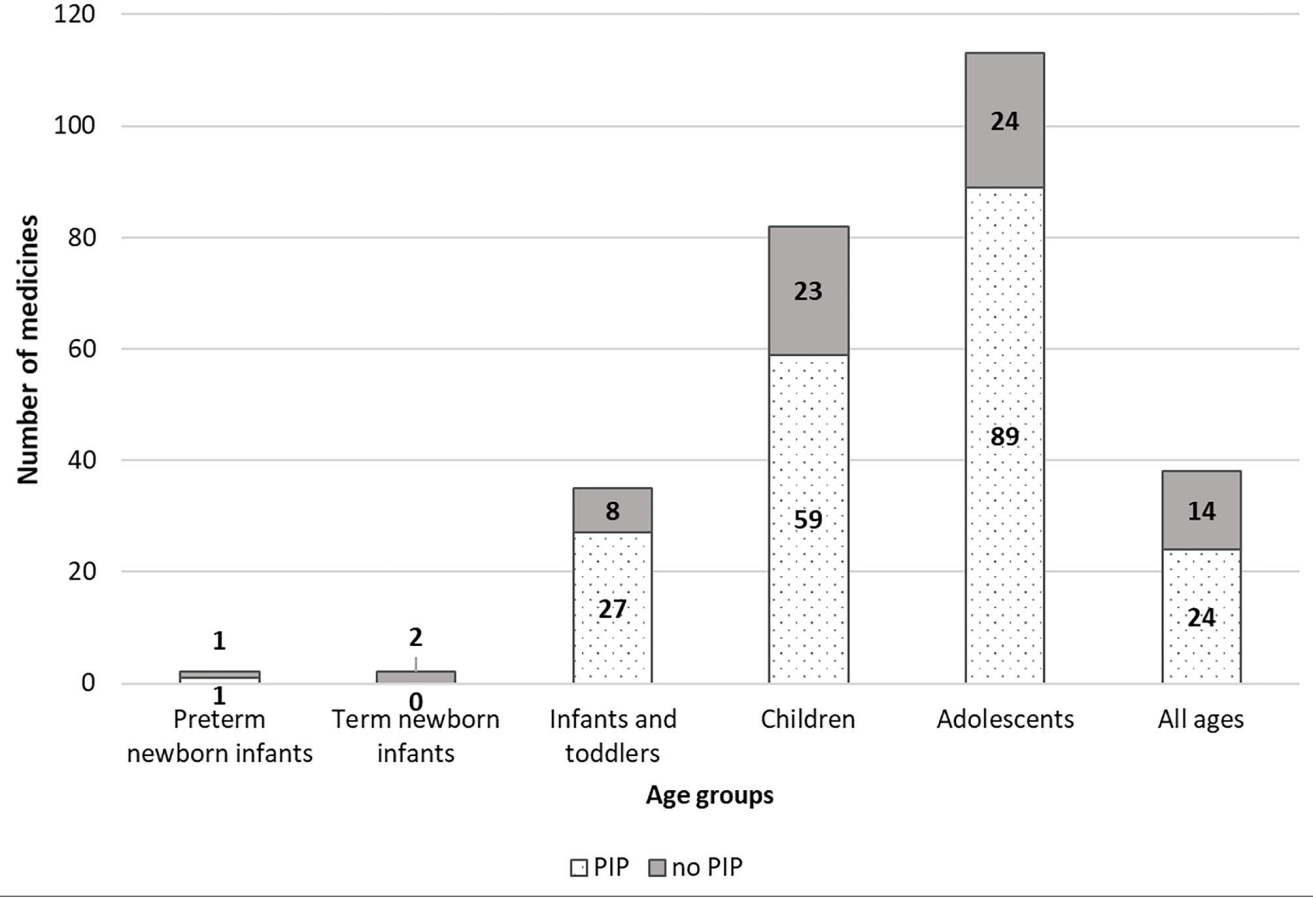
EMA paediatric medicines distributed by age groups, and PIP/no-PIP group.

**Figure 4:**
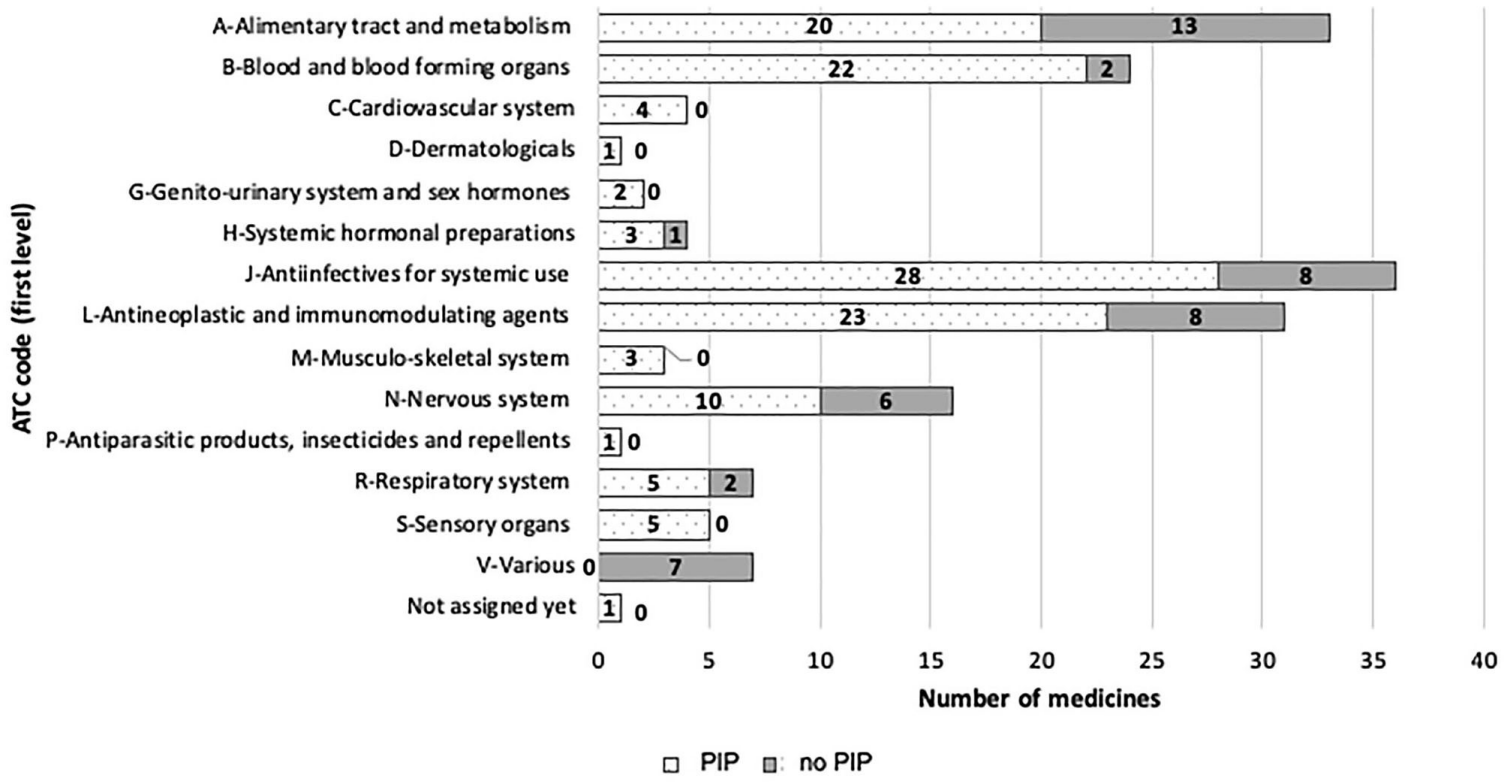
EMA paediatric medicines divided by ATC code (first-level), and PIP/no-PIP group.

On a total of 296 p-MPs, 175 have been granted a new paediatric indication, 128 in the PIP group and 47 in the no-PIP group. Of these, 126 have received a new indication and 49 an extension of a previous approved indication (from adults or other paediatric age), also associated with a new dosage calculation (3) or a new formulation (7). Details are reported in Table 2, Figures 3, 4 and described here.

**Table 2:** PIP and no-PIP paediatric medicines characteristics.

	**PIP GROUP (p-MPs 136/p-ASs 130)**			**no-PIP GROUP (p-MPs 160/p-ASs 86)**		
	**art. 7-new products**	**art. 8-in patent products**	**art. 30-off patent products**	**Generics (art. 10.1)**	**Biosimila (art. 10.4)**	**Hybrid and well-established use (art. 10.3, art 10a, art 10b, art 10c)**
**p-MPs**	92	38	6	71	31	58
p-ASs (new)^*^	88	34	6	0	0	47
**Biol/ATMPs**	53	21	0	0	0	11
**p-OMPs**	35	6	0	0	0	16
AM^**^	65	2	0	3	19	11
CA^***^	23	1	0	0	0	9

* *ASs granting new paediatric indication, dosage, age group*;

** *additional monitoring*;

*** *conditional approval, including also exceptional circumstances and accelerated assessment*.

#### Paediatric Age Groups

Figure 3 shows the number of paediatric medicines authorised for each age group, as defined by the ICH-E11 guideline (17). It is noteworthy that, notwithstanding that the number of PIPs including neonates and infants has increased in the last 10 years (9), the number of p-MPs approved for preterm and term new-born and infants remains very low, and only 38 medicines cover the all age groups of the paediatric population, 24 (19%) in the PIP group and 14 (30%) in the no-PIP group.

#### ATC Category

Paediatric drugs belong to **14 ATC** first-level categories. The percentage of paediatric drugs for each therapeutic area significantly varies among ATC codes: J-ATC (anti-infectives for systemic use) represents the group with the highest ratio (21%) of the total number of authorised medicines, while D-ATC (D—Dermatologicals) represents the lowest ratio.

In the period 2007–2019, 31 oncology p-MPs were approved, whereas there were only 17 in the 176 previous period. Figure 4 provides additional details.

#### Biological Drugs

Biological drugs have been approved for treatment of rheumatologic diseases (including juvenile idiopathic arthritis and Lupus Erythematosus) and for treatment of hepatitis C and HIV infection. In the no-PIP group, paediatric indication extension to cover all the paediatric ages has been granted to the adults' products Hizentra® and Privigen® for treatment of immunologic deficiency syndromes.

In the PIP group, three new approved advanced therapies have been granted: Zynteglo®, for beta-thalassaemia, Kymriah® for treatment of Lymphoma and Large B-Cell and Luxturna® indicated for the treatment of patients with vision loss due to inherited retinal dystrophy.

#### Drugs for Orphan Disease

57/175 medicines are p-OMPs. Six of them are for neonates and premature newborns, all except one belonging to the no-PIP group. Other interesting drugs in both PIP and no-PIP groups include medicines for neurological disease (neonatal apnoea, juvenile epilepsy, and optic hereditary atrophy), inborn errors of metabolism and cancer. Of these, many drugs have been repurposed from previous old and adults not orphan indications.

#### Paediatric Studies

The analysis was performed on the studies presented in the MA dossiers at the time of the first MA submission. A total of 530 paediatric studies were part of the MA dossiers, of which 454 (86%) were granted within a PIP and 76 (14%) were granted without a PIP.

The main difference between the medicines of the PIP group and those of the no-PIP group was the number of paediatric studies by each drug. In the no-PIP group, the ratio of studies/product corresponds to 1.6 compared to 3.3 in the PIP group. In addition, in the no-PIP group only 34% of medicines have a complete paediatric developmental plan. This limitation is also evident in the case of p-OMPs approved under the Directive 2001/83/EC procedure. Details of paediatric studies are reported in Tables 3, 4.

**Table 3:** Paediatric studies by study type.

**Study type**	**Studies in the PIP group**	**Studies in the no-PIP group**	Studies in the p-OMP PIP group^*^	Studies in the p-OMP no-PIP group^*^
PK/PD	161	18	48	3
Efficacy/safety	178	21	71	9
PK/PD/Efficacy/Safety	89	27	34	8
Observational/Metanalysis	10	5	5	4
Extrapolation/Modelling/simulation	18	5	5	0
Total	456	76	163	24

* *p-OMPs have also been counted in the previous columns*.

**Table 4:** Paediatric studies characteristics.

**Studies by MP**	**p-MPs in the PIP group (128–100%)**	**p-MPs in the no-PIP group (47–100%)**	*p-*value^*^	p-OMPs in the PIP group^**^ (41–100%)	p-OMPs in the no-PIP group^**^ (16–100%)	*p-*value^*^
Study population including only children	77 (60%)	19 (40%)	0.020	24 (59%)	6 (38%)	0.153
All 3 phases studies	89 (70%)	16 (34%)	<0.001	34 (83%)	4 (25%)	<0.001
No. of studies by approved drug	3.3	1.6	–	4.0	1.5	–

* *Chi-square test*;

** *p-OMPs have also been counted in the previous columns*.

#### Comparison of p-MPs and p-ASs Approved Before and After the Paediatric Regulation Approval

To summarise, a comparison was finally made on the two Centralised EMA procedure periods, with reference to some selected indicators of progress in the approval of paediatric medicines. Results are presented in Table 5.

**Table 5:** Main indicators before and after the Paediatric Regulation.

**Indicators**	**1996–2006**	**2007–2019**	**1996–2019**
No. of new p-ASs	106	175	281
No. of new p-ASs/year	10.6	14.6	12.7
p-ASs/ASs rate	45%	29%	33%
p-OMPs/p-MPs	13/109	57/296	70/405
No. of medicines covering the whole paediatric population	18 (17%)	38 (22%)	56 (19.5%)
No. of ATC first-level categories covered	14	14	14
No. of paediatric antineoplastic and immunomodulating agents	17 (16%)	31 (18%)	48 (17%)
No. of neonates and infants MPs	26 (25%)	37 (21%)	63 (23%)
% of approved drugs including a whole clinical developmental plan (three phases)	51.6%	60%	55.8%
% of only children trials	52%	53%	52.5%
% of medicines approved with no paediatric trials	8.1%	9.1%	8.6%

## Discussion

The current paper demonstrates that after 12 years of the Paediatric Regulation being in force, the trend for paediatric medicines is stable compared to p-MPs authorised before 2007. However, when considering the number of ASs, we noted a decreased percentage of p-ASs compared to the period before 2007. More specifically, the average annual increase of both p-MPs and ASs is different and significantly lower in the case of p-ASs. Moreover, among the p-ASs, only a limited percentage represent “new” paediatric medicines. In fact, our data demonstrate that more than 1/3 of products are generic or biosimilar products not including new paediatric indications, dosages or age groups.

Another interesting result of our analysis derives from the comparison of two different groups of EMA approved paediatric medicines. The first group is represented by the medicines that have been granted a PIP according to the Paediatric Regulation procedure. This group represents <50% of all paediatric medicines. The second group includes medicines granted a centralised MA under Directive 2001/83/EC without submitting a PIP (the no-PIP group). Of these, 106 (corresponding to 41 ASs) are generics and biosimilars, and do not include new paediatric characteristics, while the remaining medicines were repurposed products which included new paediatric indications, age extension or new dosage.

The new p-MPs approved under the Paediatric Regulation provisions include advanced therapies, with more biological products than chemical ones, demonstrating that innovative medicines are increasingly promoted for children when developed in the framework of an *ad hoc* PIP. Most biologicals with a paediatric indication belong to the class of antineoplastic and immunomodulating agents, even if there was only a limited increase of neonatal and oncology p-MPs, and no increase in neurology p-MPs was found compared to that reported in the research conducted up to 2006 (1).

As in the previous report (1), the ratio of p-OMPs/total OMPs is higher than in case of not orphan MPs, corresponding to 40% and globally the number of p-OMPs has raised to 57 approved paediatric orphan indications from the previous 13 indications.

With reference to the expected increase of paediatric trials and studies, it is encouraging that 70% of p-MPs that received a PIP approval include the full range of the 3 phases of clinical studies in the MA dossier. This allows us to conclude that receiving a PIP allows MA holders to provide good and complete data on efficacy and safety in children, even if a high number of trial results are still incomplete at the time of submission of the MA. This also supports data from the 10-year review of the Paediatric Regulation carried out by the EC in 2017 (9) that recognized a significant increase in the number of studies supporting a paediatric indication.

On the other hand, this ratio is much higher than that one observed in p-MPs approved outside the Paediatric Regulation (34%). The no-PIP group is globally less supported by robust results of paediatric studies, even in the case of orphan paediatric indications (only 24 studies for a total of 16 products). Some drugs, following a hybrid application, were introduced on the EU market without submitting the results of any new study. Additional considerations should be given to the limited presence in the MA dossiers of paediatric studies, such as extrapolation and modelling & simulation studies, which are currently promoted at scientific and regulatory level in support of traditional studies in order to reduce the burden of the drug development process and accelerate the time to the market (18–21).

Finally, we considered the very high number of medicines submitted to the additional monitoring procedure in both the PIP and no-PIP groups. It is questionable whether this represents a consequence of incomplete data at the time of the MA. Same consideration can be done with reference to fast track approvals, which have been granted to almost 20% of paediatric medicines. This aspect has never been analysed in any paediatric medicine publication or official reports with reference to both the PIP and no-PIP groups, requiring a follow-up action to collect additional information on the paediatric medicines post-marketing phase.

Moreover, from the analysis of the group of paediatric medicines approved outside the Paediatric Regulation (no-PIP group), following hybrid or well-established use procedures, we derived additional interesting results. The majority of medicines in this group represents old *off-patent* medicines repurposed for paediatric use and allowing (a) therapeutic indication extension to uncovered paediatric ages including neonate (6 on 7 neonates, preterms or infants approved MPs are from no-PIP group), or (b) new indication, and (c) implementation of p-OMPs (16 orphan indication of interest for children including for the treatment of rare cancers and for genetic and neonatal diseases).

We can conclude that old medicinal products could give in the field of paediatric medicines a relevant contribution also reducing the off-label use of adults' medicines, largely affecting children in many paediatric ages and serious diseases. This value is also recognised at regulatory level since medicines in the no-PIP group share with medicines in the PIP group an high rate of MA granted under exceptional circumstances (accelerated assessment and conditional approval) that are special regulatory procedures granted if there is the need to go rapidly to the market for reason of patients serious conditions and needs.

On the basis of these considerations, we conclude that the expectation of the Paediatric Regulation to provide the paediatric population with safe access to older and innovative drugs has been substantially met. However, some limitations have been also underlined that correspond to what also discussed in recent publication and, in particular, in the analyses done by EMA and the European Commission concluded with the proposal to address modification of the Paediatric Regulation.

In particular, this paper underlines that a significant number of *off-patent* drugs were approved for paediatric use outside of the obligation to submit a PIP and to address the PDCO opinion. For these products, a PUMA application is foreseen in the Paediatric Regulation, but our data confirm that sponsors prefer to apply under the simplified procedure of Directive 2001/83/EC where an *off-patent* drug is concerned. These medicines also cover relevant therapeutic needs (including neonates, oncology, and orphan diseases) and have a special role in practically reducing the off-label use of adults' medicines. However, in many cases these drugs are approved for a completely new paediatric indication but the clinical evidence accumulated before the approval is very scarce and significantly inferior to what existing in case of the drugs that have been granted a PIP.

These and other aspects should be part of the ongoing discussions and relevant initiatives in the regulatory field such as the **Action Plan on Paediatrics** (10) and the **open consultation on EU Pharmaceutical Strategy** (11), from which it is anticipated that revision of both paediatric and orphan drug regulations will be implemented.

We can consider these initiatives as a great opportunity to further implement the Paediatric Regulation results and to identify the right framework to support the research for more safe and innovative paediatric medicines in Europe.

## Data Availability Statement

The data analyzed in this study is subject to the following licenses/restrictions: Dataset can be provided upon request. Requests to access these datasets should be directed to scientificsecretariat@teddynetwork.net; https://www.teddynetwork.net.

## Author Contributions

AC: coordination, outline, data analysis, and writing. MF: outline, revision, and suggestion for further implementation. DB, FB, SW, and VG: revision and suggestion for further implementation. GR: statistical analysis. MT: data analysis, writing, figures, tables, drafting. All authors contributed to the article and approved the submitted version.

## Conflict of Interest

The authors declare that the research was conducted in the absence of any commercial or financial relationships that could be construed as a potential conflict of interest.
